# MiR-942 Mediates Hepatitis C Virus-Induced Apoptosis via Regulation of ISG12a

**DOI:** 10.1371/journal.pone.0094501

**Published:** 2014-04-11

**Authors:** Darong Yang, Xianghe Meng, Binbin Xue, Nianli Liu, Xiaohong Wang, Haizhen Zhu

**Affiliations:** 1 Department of Molecular Medicine, State Key Laboratory of Chemo/Biosensing and Chemometrics, Hunan University, Changsha, China; 2 Research Center of Cancer Prevention & Treatment, Translational Medicine Research Center of Liver Cancer, Hunan Provincial Tumor Hospital (Affiliated Tumor Hospital of Xiangya Medical School of Central South University), Changsha, China; Temple University School of Medicine, United States of America

## Abstract

The interaction between hepatitis C virus (HCV) and human hepatic innate antiviral responses is unclear. The aim of this study was to examine how human hepatocytes respond to HCV infection. An infectious HCV isolate, JFH1, was used to infect a newly established human hepatoma cell line HLCZ01. Viral RNA or NS5A protein was examined by real-time PCR or immunofluorescence respectively. The mechanisms of HCV-induced IFN-β and apoptosis were explored. Our data showed that HLCZ01 cells supported the entire HCV lifecycle and IFN-β and interferon-stimulated genes (ISGs) were induced in HCV-infected cells. Viral infection caused apoptosis of HLCZ01 cells. Silencing of RIG-I, IRF3 or TRAIL inhibited ISG12a expression and blocked apoptosis of viral-infected HLCZ01 cells. Knockdown ISG12a blocked apoptosis of viral-infected cells. MiR-942 is a candidate negative regulator of ISG12a predicted by bioinformatics search. Moreover, HCV infection decreased miR-942 expression in HLCZ01 cells and miR-942 was inversely correlated with ISG12a expression in both HCV-infected cells and liver biopsies. MiR-942 forced expression in HLCZ01 cells decreased ISG12a expression and subsequently suppressed apoptosis triggered by HCV infection. Conversely, silencing of miR-942 expression by anti-miR-942 increased ISG12a expression and enhanced apoptosis in HCV-infected cells. Induction of Noxa by HCV infection contributed to ISG12a-mediated apoptosis. All the data indicated that innate host response is intact in HCV-infected hepatocytes. MiR-942 regulates HCV-induced apoptosis of human hepatocytes by targeting ISG12a. Our study provides a novel mechanism by which human hepatocytes respond to HCV infection.

## Introduction

Hepatitis C virus (HCV) infects about 170 million people worldwide [1]. The majority of those infected develop chronic infection, leading to chronic hepatitis, liver cirrhosis and even hepatocellular carcinoma [1], [2]. There is no vaccine for HCV. 20% to 30% of those acutely infected with HCV may clear the virus without treatment, indicating that innate and/or adaptive immune responses are capable of controlling the outcome of HCV infection. Therefore, the molecular events regulating innate intracellular antiviral responses may serve as pivotal points of control, potentially limiting host permissiveness for HCV replication.

The innate immune response to virus infection is activated when conserved pathogen-associated molecular patterns (PAMPs) generated during infection are recognized by proteins known as pattern recognition receptors (PRRs) such as Toll-like receptors (TLRs), and RIG-I-like receptors (RLRs) [3], [4]. Viral engagement of TLRs and RLRs leads to downstream signaling resulting in the activation of latent transcription factors, including IFN regulatory factors (IRFs) and nuclear factor-kB (NF-kB), and culminates in the induction of IRF3 target genes, type I IFN. In mammalian cells, IFN gene transcription is induced through distinct signaling pathways by viral infection. Virus-induced production of IFNs and the subsequent expression of IFN-stimulated genes (ISGs) are central to the innate antiviral defenses, which functions to limit viral replication [5]. RIG-I senses dsRNA, an essential intermediate in the HCV lifecycle, and may be important in the pathogenesis of hepatitis C [6], [7]. One recent study showed that HCV genome 3′ non-translated region as HCV PAMP stimulates RIG-I-dependent signaling to induce innate immunity and triggers the expression of IFN and ISG [8].

Development of infectious HCV clone (JFH1) provides a powerful tool for the analysis of host-virus interactions [9]–[11]. However, the permissive human hepatoma cell Huh7.5 may not mount an intact innate antiviral response to HCV infection, so it is difficult to explore the interaction between host cells and natural HCV infection. Antiviral responses have been studied in human hepatic-derived cell lines Huh7 and Huh7.5 cells which may differ from human hepatocytes having intact immune responses in their antiviral signaling pathways [12], [13]. It is necessary to identify other cell lines permissive for HCV infection and having intact innate antiviral defense system to explore the mechanism of how human hepatocytes respond to HCV infection.

Here we demonstrated that infectious HCV could infect HLCZ01 cells and stimulated innate immune responses in the cells through induction of IFN and triggering apoptosis of viral-infected cells. RIG-I played a key role in the induction of IFN and apoptosis by HCV in the cells. MiR-942 regulated HCV-induced apoptosis of human hepatocytes through targeting ISG12a. Induction of Noxa by HCV infection contributed to ISG12a-mediated apoptosis.

## Materials and Methods

### Ethics Statement, isolation and establishment of a novel hepatoma cell line

All the patients signed consent form to acknowledge participation in the study approved by the Review Board of Hunan Provincial Tumor Hospital. Experimental procedures were performed in accordance with the Ethics Committee of Hunan Provincial Tumor Hospital. HLCZ01 cells were isolated from liver cancer tissues and cultured as described previously [14]. Cells were isolated from the liver tumor tissue of a 60 year-old male patient. Briefly, immediately after surgical resection, the tumor tissue was stored in PBS, and dissociation of the cells was performed within one hour by two-step perfusions. Visible vessels were first perfused for 15 minutes with Liver Perfusion Medium (Invitrogen) to eliminate the blood cells. A second perfusion was performed with Liver Digest Medium (Invitrogen), until the tissue was digested. Liver tissues were cut into small pieces and shaken gently in Hepatocytes Wash Medium (Invitrogen). The cells and small pieces of liver tissue were culture with DMEM/F12 medium supplemented with 10% FBS (Invitrogen). The cell clones were picked and cultured in 6-well plate. The cells were expanded until cell growth was sufficient to fulfill the culture well. To obtain hepatoma cell line, 5×10^6^ cells were injected into NOD/SCID immunodeficiency mice. Two months later, the tumor tissues removed from the mice were cut into small pieces and cultured in DMEM/F12 medium. One clone was called HLCZ01 cell. Liver tissues from HCV-infected patients were collected from 2009 to 2012 in Hunan Provincial Tumor Hospital.

### Cells culture, reagents and plasmids

Huh7.5 cells were kindly provided by Charles Rice (Rockefeller University, New York, NY) [15]. The expression vector pcDNA3.1/V5-His was from Invitrogen (Carlsbad, CA). The shRNAs targeting TRAIL, RIG-I shRNA targeted human RIG-I, and negative control shRNA were purchased from Santa Cruz Biotechnology (Santa Cruz, CA). Monoclonal antibodies against human CD81 (5A6) and IRF-3, rabbit polyclonal anti-RIG-I antibody (H300), goat polyclonal anti-Noxa antibody (N-15), goat anti-mouse or goat anti rabbit IgG-HRP secondary antibody were from Santa Cruz Biotechnology. Monoclonal antibodies against β-actin and PUMA (10D4G7) were from Sigma. Rabbit polyclonal anti-ISG12a antibody (ab14695) was from ABCAM. Rabbit monoclonal anti-Cytochrome c antibody (136F3) and anti- PARP antibody (46D11) were from Cell Signaling Technology. Supersignal West Pico Chemiluminescent Substrate was from Pierce. 2′-O-me-anti-miR-942 (5′-CACAUGGCCAAAACAG AGAAGA-3′) and 2′-O-me-control-miR (5'-AAGGCAA GCUGACCCUGAAGU-3′) were from Takara. Mouse monoclonal anti-NS5A (HL1126) was a gift from Chen Liu (University of Florida, Gainesville, FL).

### HCV constructs and viral particle generation

pJFH-1 and pJFH-1/GND plasmids were gifts from Takaji Wakita (National Institute of Infectious Diseases, Tokyo, Japan) [10]. The linearized DNA was purified and used as a template for in vitro transcription using MEGAscript kit (Ambion, Austin, TX). In vitro transcribed genomic JFH-1 or JFH-1/GND RNA was delivered into Huh-7.5 cells by electroporation. The transfected cells were transferred to complete DMEM and cultured for the indicated period. Cells were passaged every 3–5 days, and corresponding supernatants were collected and filtered with a 0.45-µm filter device. The viral titers were expressed as focus-forming units per milliliter, determined by the average number of NS5A-positive foci detected in Huh-7.5 cells [11].

### Real-time PCR assays

The primers targeted HCV, IFN-β, TRAIL, DR4, DR5, RIG-I, 1-8U and G1P3 have been reported previously and real-time PCR were performed as described previously [16]. The cDNA of miR-942 was synthesized from total RNA using stem-loop RT primer (5′-GTCGTATCCAGTGCAGGGTCCGAGGTATTCGCAC TGGATACGACCACAT-3′), and miR-942 was quantized by real-time PCR using primers 5-GCGCGCTCTTCTCTGTTTTGGC-3′ and 5′-GTGCAGGGTCCGAGGT-3′. The internal control was U6. The cDNA of U6 was synthesized from total RNA using stem-loop RT primer (5′-CGCTTCACGAATTTGCGTGTCAT-3′), and U6 was quantized by real-time PCR using primers 5′-GCTTCGGCAGCACATATACAAAAT-3′ and 5′-CGCTTCACGAATT TGCGTGTCAT-3′. Fold variations were calculated after normalization to U6. ISG12 was quantized by real-time PCR using primers 5′-TGCCATGGGCTTCACTGCGG-3′ and 5′-CTGCCCGAGGCAACTCCACC-3′. The primers for human GAPDH were 5'-GCA CCGTCAAGGCTGAGAAC-3' and 5'-TGGTGAAGACGCCAGTGGA-3' (Takara).

### Plasmid construction

The oligonucleotides encoding 19-mer hairpin sequence specific to the targets mRNA were incorporated into the pSilencer-neo plasmid (Ambion). The sequences of ISG12a shRNAs targeting two regions of ISG12a were 5′-AAGTTCATCCTGGGCTCCATT-3′ and 5′-AATTAACCCGAGCAGGCATGG-3′. The sequences of IRF3 shRNAs targeting three regions of IRF3 were 5′-AATACTGTGGACCTGCACATT-3′, 5′- AAGAGGCTCGTGATGGTCAAG-3′ and 5′- AAGGCCTACCTGCAGGACTTG-3′. The sequences of Noxa shRNAs targeting two regions of Noxa were 5′-AAGTAATTATTGACACATTTC-3′ and 5′-AAGTCGAGTGT GCTACTCAAC-3′. Precursor of miR-942 was amplified from Huh7 cells and inserted into pcDNA3.1/V5-His. The primers for miR-942 are 5′-GCATGGATCCGCTTTA ACAATGGTTCCTCCG-3′ (F) and 5′-GCCGGTCTAGAAGCACCTTTTGTTTCTATT ATCACG-3′ (R). The 3′UTR of the human ISG12a gene (GI:194272170) was PCR amplified using the following primers: ISG12a (F), 5′-TTAATAATCTAGACTCCCTG CCCCTCGCCCTGCA-3′, and ISG12a (R), 5′-GCGCCGGGTCTAGAGAAGAGTTGC AACAATTCATC-3′. The amplified product was cloned downstream of the Renilla luciferase in pGL3 control vector (Promega) and pGL3-ISG12aUTR luciferase construct containing wild type ISG12a 3′UTR was obtained. The antisense sequence of 3′UTR of ISG12a was introduced into pGL3 control vector and the pGL3-ISG12aUTR (Mut) construct was obtained. All constructs were sequenced, and transfected into cells using Lipofectamine 2000 reagents (Invitrogen).

### Immunofluorescence

The protocol has been reported previously [17].

### Western blot analysis

The procedure was reported previously [17]. Briefly, cells were washed with PBS and lysed in RIPA buffer (150 mM sodium chloride, 1% Nonidet P-40, 0.5% sodium deoxycholate, 0.1% SDS and 50 mM Tris-HCl [pH 8.0] supplemented with 2 µg/mL of aprotinin, 2 µg/mL of leupeptin, 20 µg/mL of phenylmethysulfonyl fluoride, and 2 mM DTT). Forty micrograms of protein were resolved by SDS/PAGE, transferred to a PVDF membrane and probed with appropriate primary and secondary antibodies. The bound antibodies were detected by ECL reagent (Pierce, Rockford, IL) according to the manufacturer's instruction.

### Flow cytometry

The procedure has been reported previously [14]. Samples were analyzed on a FACS Caliber Cytometer (BD Pharmingen). For all FACS, the data of Annexin-V positive sections (upper right and lower right) were quantified as a basis for the bar graphs. The data were analyzed with CellQuest software.

### Statistical Analysis

Student *t*-test was applied to determine statistical significance. Bars represented S.D. ^*^ *P*<0.05, ^**^ *P*<0.01, ^***^ *P*<0.001 verse control.

## Results

### A newly developed human hepatocellular carcinoma cell line, HLCZ01, is susceptible to HCV JFH-1 isolate

**Figure 1:**
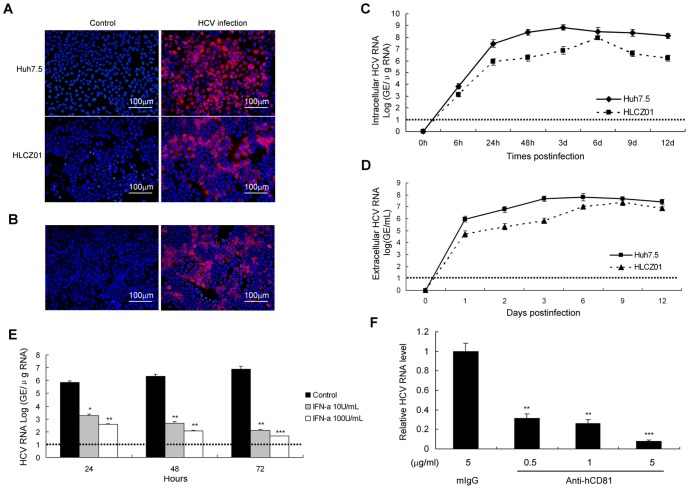
HCV infection of HLCZ01. (**A**) Filtered supernatant of JFH1 RNA-transfected Huh7.5 cells was inoculated with naive Huh7.5 or HLCZ01 cells. Cells were immunostained with mouse monoclonal anti-NS5A antibody at day 6 after inoculation. DAPI was used for nuclei counterstaining. (**B**) Naïve HLCZ01 cells were incubated for 3 days with filtered, conditioned media collected from HCV-infected HLCZ01 cells and immunostained for NS5A expression. (**C**) Viral RNA kinetics determined by real-time PCR in HLCZ01 and Huh7.5 cells infected by JFH1 virus at MOI of 0.1. HCV RNA in HCV-infected cells was determined by real-time PCR. The viral replication is represented by HCV genome equivalence (GE)/µg total cellular RNA. (**D**) Viral RNA in the supernatant of HCV-infected HLCZ01 and Huh7.5 cells determined by real-time PCR. The viral RNA is calculated as GE per milliliter medium using a standard curve generated by in vitro transcribed full-length JFH1 RNA. (**E**) IFN inhibits HCV RNA replication in HLCZ01 cells in a dose-dependent manner. (**F**) Anti-CD81 antibody blocked HCV infection in HLCZ01 cells. HLCZ01 cells were pretreated with anti-CD81 antibody for 2 hours before viral inoculation. Viral RNA was analyzed by real-time PCR at day 3 pi. If not stated otherwise bar graphs represent means of three independent experiments. Horizontal dashed lines indicate the low limit of quantification (LLOQ) of the assay.

The recent development of infectious HCV cell culture system taking advantage of a genotype 2a patient isolate, JFH1, provides a tool for the study of viral lifecycle and the interaction between virus and host cells. JFH1 RNA was transfected into Huh-7.5 cells. Cell supernatant harvested at day 17 after transfection was then used to infect naive Huh-7.5 cells. After 6 days of infection, the majority of the cells were infected as determined by detection of viral NS5A protein in the cells (Figure 1A).

Huh7.5 cells lack a functional RIG-I signaling pathway because of mutant RIG-I (T55I) in the cells and may not mount an intact innate immune response system in response to HCV infection [12]. To better understand the interaction between virus and host cells, we have attempted to replicate JFH-1 virus in other hepatoma cells. We established a new cell line, referred to as HLCZ01 from hepatocellular carcinoma tissue of a male patient. Histologically, tumor cells have a characteristic histological feature of hepatocellular carcinoma (Figure S1A). The cells express the markers of human hepatocytes such as human albumin protein and α-1-antitrypsin (Figure S1B). To determine whether HLCZ01 cells are permissive for HCV infection, we inoculated HLCZ01 cells with JFH-1–containing cell culture supernatant from JFH-1 RNA-transfected Huh-7.5 cells. Cells were harvested for immunostaining using monoclonal anti-HCV NS5A antibody at 6 days after viral inoculation. HCV-infected HLCZ01 cells were clearly detected (Figure 1A). To prove that HCV-infected HLCZ01 cells indeed release infectious virus, we inoculated naïve HLCZ01 cells with the filtered supernatant collected from HCV-infected HLCZ01 cells and the cells were harvested for immunofluorescence staining for NS5A protein assay 3 days postinfection (pi). Naïve HLCZ01 cells inoculation with the supernatant of HCV-infected HLCZ01 cells were positive for HCV NS5A protein (Figure 1B), clearly indicating that HCV-infected HLCZ01 can release infectious virus.

To get the HCV infection kinetics in HLCZ01 cells, we inoculated HLCZ01 and Huh7.5 cells with JFH1 virus at MOI of 0.1, and then examined the viral RNA kinetics in both cells and culture medium. The viral RNA could be easily detected inside the cells and the supernatant of viral-infected HLCZ01 cells after 24 hours of infection. The viral kinetics inside the cells and culture medium was comparable (Figure 1C and 1D). The viral replication efficiency was slightly lower in HLCZ01 cells than in Huh7.5 cells.

To distinguish the difference between the inputted viral RNA and newly synthesized viral RNA, we treated virus-infected HLCZ01 cells with IFN-α. Viral RNA replication in HLCZ01 cells was suppressed by IFN-α in a dose-dependent manner (Figure 1E). We blocked viral entry using anti-CD81 antibody, because CD81 is a critical HCV receptor [18], [19]. This antibody has been shown to block HCV and CD81 binding [20]. Moreover, the expression of CD81 in HLCZ01 and permissive Huh7.5 cells was comparable (Figure S1C). When HLCZ01 cells were treated with anti-CD81 antibody before viral inoculation, viral infection efficiency was markedly decreased (Figure 1F). All the data suggested that HLCZ01 cells are susceptible to JFH1 virus and release infectious virus.

### HCV infection induces type I IFN and ISGs in HLCZ01 cells

**Figure 2:**
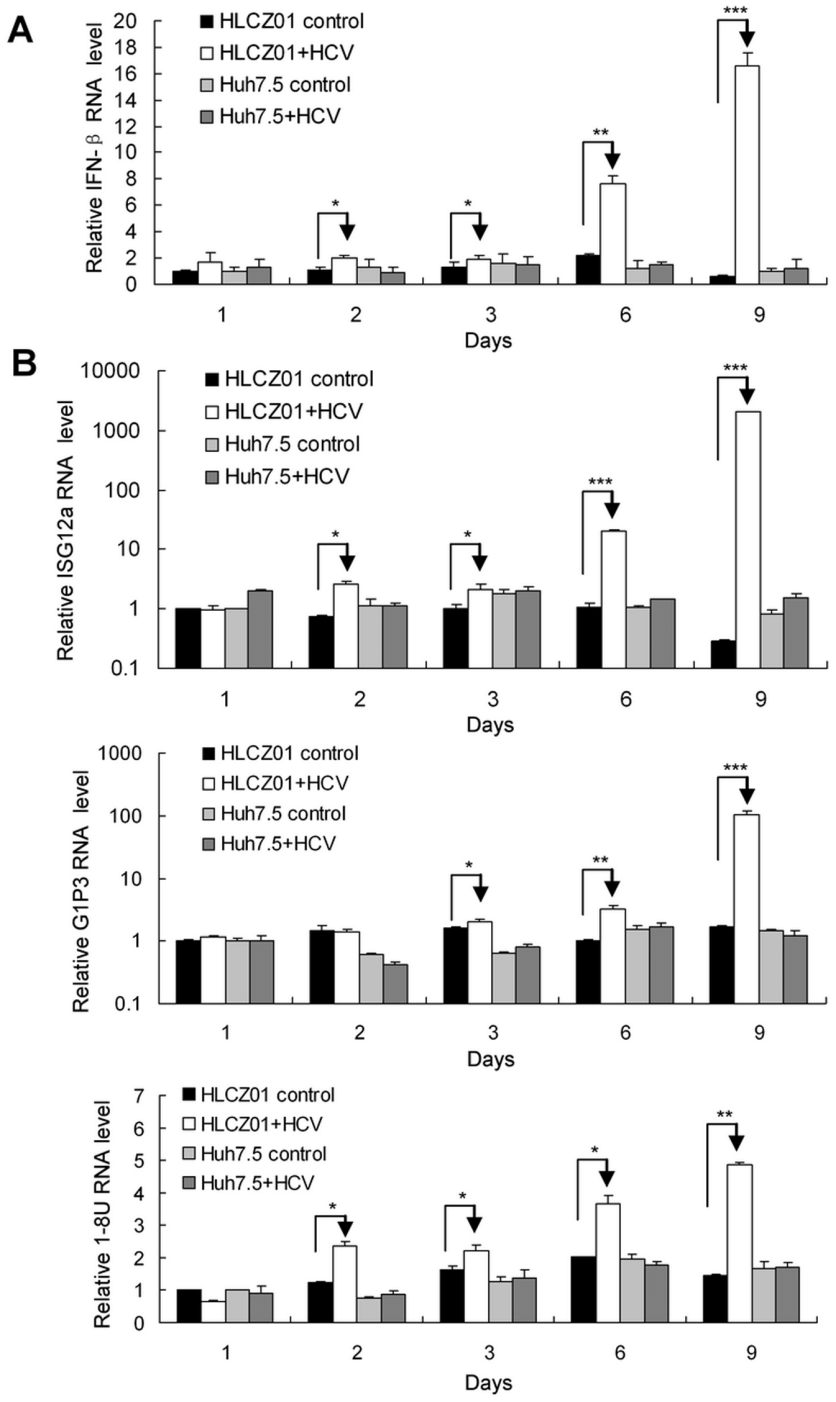
HCV infection induces type I IFN and ISGs in HLCZ01 cells. (**A**) Kinetics of IFN-β in HCV-infected HLCZ01 cells. HLCZ01 and Huh7.5 cells were infected with JFH1 virus at MOI of 0.1. Cells were harvested for total RNA extraction at different time points. The kinetics of induction of IFN-β was analyzed by real-time PCR and normalized with GAPDH. (**B**) Kinetics of ISG12a, G1P3 and 1–8U in viral-infected HLCZ01 cells. HLCZ01 and Huh7.5 cells were treated as described in part A. The expression of ISG12a, G1P3 and 1–8U mRNA was analyzed by real-time PCR and normalized with GAPDH respectively. If not stated otherwise bar graphs represent means of three independent experiments.

Production of type I IFN by virus-infected cells is a central event in antiviral response of host cells. To test whether HCV can induce innate immune response in HLCZ01 cells, we infected HLCZ01 cells with JFH1 virus and monitored IFN-β expression. The expression of IFN-β in HLCZ01 cells could be easily detected by real-time PCR assay (Figure 2A). The IFN-β protein level in the supernatant of HCV-infected HLCZ01 was too low to be detected. IFN triggers intracellular innate antiviral response to limit viral replication and functions through activation of ISGs. Our previous study demonstrated that ISGs including 1–8U and G1P3 play an important role in the establishment of intracellular antiviral state [5], [21]. To confirm the IFN-induced antiviral pathway is functional in HLCZ01 cells, we examined the expression of ISGs in HCV-infected HLCZ01 cells. The expression of G1P3, 1–8U and ISG12a could be detected in viral-infected HLCZ01 cells (Figure 2B). These data indicated that HCV induces type I IFN and ISGs in HLCZ01 cells.

### HCV infection triggers apoptosis of HLCZ01 cells

**Figure 3:**
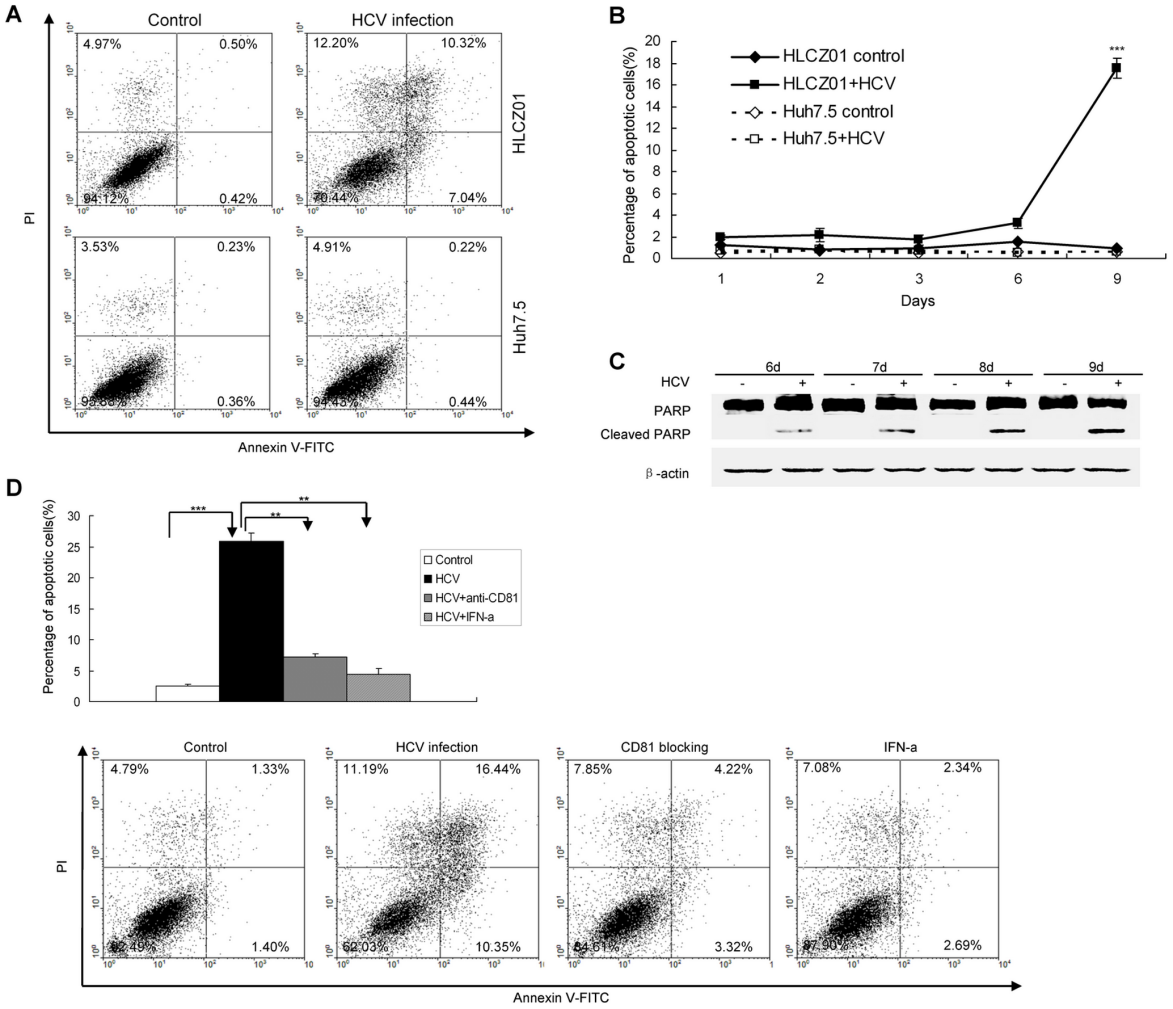
HCV infection triggers apoptosis of HLCZ01 cells. (**A**) Annexin V expression determined by flow cytometry. HLCZ01 and Huh7.5 cells were infected with HCV at MOI of 0.1. The cells were harvested at day 9 pi and subjected to Annexin V analysis determined by Flow cytometry. The data are one representative of three independent experiments. (**B**) Kinetics of apoptosis in HCV-infected HLCZ01 cells. HCV-infected HLCZ01 and Huh7.5 cells were harvested for Annexin V expression determined by flow cytometry. The percentage of apoptotic cells is plotted. The data represent the means of three experiments. (**C**) Confirmation of HCV-induced apoptosis in HLCZ01 cells by western blot. HLCZ01 cells were infected with HCV at MOI of 0.1. Cells were collected and PARP cleavage was detected by western blot. Blots are representative of three independent experiments. (**D**) Blocking viral entry by anti-CD81 antibody or suppression of HCV replication by IFN reduces apoptosis of HLCZ01 cells. HLCZ01 cells were treated with anti-CD81 antibody or 100 IU/mL IFN before viral inoculation. The cells were harvested at day 9 pi for Annexin V expression determined by Flow cytometry. The graph shows the percentage of apoptotic cells, which represents the mean of 3 independent experiments.

Interestingly, some of HLCZ01 cells infected with JFH1 virus died. When the cells were stained with DAPI for nuclear morphology, HCV-infected HLCZ01 cells showed nuclear shrinkage and fragmentation, a feature of cellular apoptosis (Figure S2). To examine whether cell death involves apoptosis in viral-infected HLCZ01 cells, we infected the cells with JFH1 virus and performed Annexin V staining with flow cytometry. Higher level of Annexin V was detected in viral-infected HLCZ01 cells compared with the control (Figure 3A). These data suggested that HCV causes apoptosis of HLCZ01 cells.

To further confirm that apoptosis of HLCZ01 cells is directly related to HCV infection, we performed a series of experiments. First, we examined the apoptotic kinetics by determining Annexin V expression after viral infection. Cell death was time dependent (Figure 3B and 3C), corresponding to viral replication kinetics in the cells (Figure 1C). Secondly, blocking viral infection with anti-CD81 antibody markedly decreased virus-induced apoptosis (Figure 1F, Figure 3D). Finally, inhibition of HCV replication by IFN-α protected HLCZ01 cells from apoptosis (Figure 3D). These data clearly suggested that HCV infection triggers apoptosis of HLCZ01 cells.

### Induction of IFN-β and apoptosis by HCV infection is mediated through RIG-I and IRF-3

**Figure 4:**
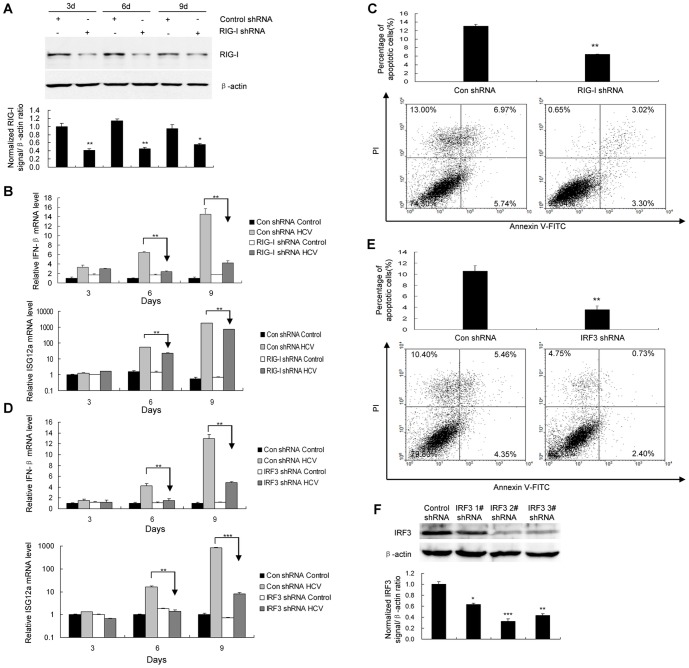
Induction of IFN-β and apoptosis by HCV infection is mediated through RIG-I and IRF-3. (**A**) Confirmation of RIG-I knockdown with RIG-I-specific shRNA in HLCZ01 cells. RIG-I shRNA or control shRNA was delivered into HLCZ01 cells followed by HCV infection. RIG-I protein was determined by western blot. (**B**) Knocking down RIG-I inhibits the induction of IFN-β and ISG12a by HCV infection in HLCZ01 cells. HLCZ01 cells were treated as described in part A. The expression of IFN-β or ISG12a mRNA was examined using real-time PCR and normalized with GAPDH. (**C**) RIG-I knockdown blocks HCV-induced apoptosis of HLCZ01 cells. RIG-I shRNA or control shRNA was delivered into HLCZ01 cells followed by HCV infection for 9 days. Apoptosis of HLCZ01 cells was examined using flow cytometry. (**D**) IRF-3 directly regulates IFN-β and ISG12a mRNA expression in HCV-infected HLCZ01 cells. HLCZ01 cells were transfected with control shRNA or IRF-3–specific shRNA for 24 hours, followed by JFH-1 infection. The expression of IFN-β and ISG12a was detected using real-time PCR analysis and normalized with GAPDH. (**E**) Knockdown of IRF-3 reduces apoptosis of HCV-infected HLCZ01 cells. HLCZ01 cells were treated as described in part D. The cells were harvested for flow cytometry. (**F**) The knockdown efficiency was examined by western blot analysis using anti–IRF-3 antibody. The abbreviation “con” is for “control” in the figures. If not stated otherwise bar graphs represent means of three independent experiments.

RIG-I plays an important role in dsRNA-induced innate antiviral responses [22], [23]. To determine whether RIG-I plays a role in the induction of IFN by HCV infection in HLCZ01 cells, we performed shRNA knockdown experiments. RIG-I shRNA significantly knocked down RIG-I protein (Figure 4A). When RIG-I shRNA was delivered into HLCZ01 cells, followed by HCV infection, IFN-β and ISG12a were reduced in comparison with the control (Figure 4B). Similar to IFN-β and ISG12a reduction, silencing of RIG-I significantly decreased HCV-induced apoptosis (Figure 4C). These data indicated that RIG-I is responsible for the induction of IFN-β and apoptosis of human hepatocytes in response to HCV infection.

IRF-3 is an important mediator for IFN induction in response to viral infection. We hypothesized that IRF-3 may serve as a key factor to control IFN induction and cellular apoptosis, the two biological processes beneficial for host antiviral defense. When IRF-3 shRNA was transfected into HLCZ01 cells, followed by HCV infection, IFN-β and ISG12a induction were markedly reduced as determined by real-time PCR (Figure 4D). Consistent with the observation of reduction of IFN-β and ISG12a, knockdown IRF-3 protected cells from HCV-induced apoptosis, as measured by flow cytometry (Figure 4E). The efficiency of IRF-3 shRNA knockdown was confirmed by western blot analysis (Figure 4F). These data implicate the direct role of IRF-3 in HCV-induced IFN expression and apoptosis, thereby causing antiviral activity via noncytolytic and cytolytic mechanisms respectively.

### Apoptosis induction by HCV infection involves ISG12a which relies on TRAIL-mediated signaling

**Figure 5:**
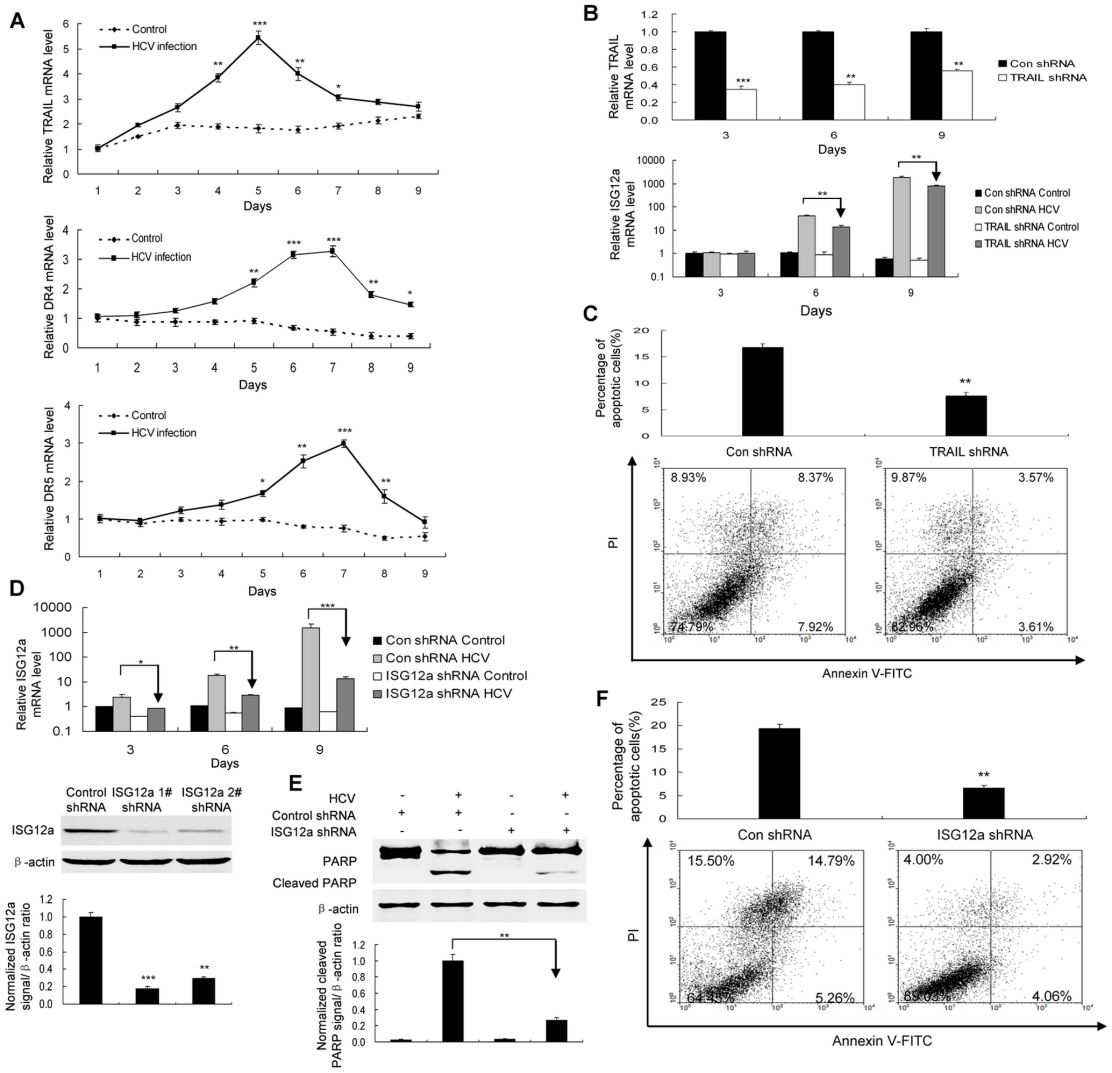
Apoptosis induction by HCV infection in HLCZ01 cells involves ISG12a which relies on TRAIL-mediated pathway. (**A**) HCV infection induced TRAIL and its receptors DR4 and DR5. HLCZ01 cell were infected by HCV at MOI of 0.1 for different time periods. TRAIL, DR4 and DR5 mRNA was examined by real-time PCR and normalized with GAPDH. (**B/C**) Silencing of TRAIL inhibited the induction of ISG12a and blocked apoptosis of viral-infected cells. TRAIL shRNA was delivered into HLCZ01 cells, followed by HCV infection. (B) TRAIL and ISG12a mRNA was detected by real-time PCR and normalized with GAPDH. (C) Cells were collected for flow cytometry analysis. (**D**) Confirmation of ISG12a knockdown with ISG12a-specific shRNA in HLCZ01 cells. The plasmid pSilencer-ISG12a shRNA was delivered into HLCZ01 cells. ISG12a mRNA and protein was detected by real-time PCR and western blot receptively. (**E/F**) ISG12a knockdown prevented HCV-infected HLCZ01 cells from apoptosis. The plasmid pSilencer-ISG12a shRNA was delivered into HLCZ01 cells. The cells were infected with HCV for 9 days. PARP cleavage was examined by western blot (E). The cells were examined by flow cytometry (F). If not stated otherwise bar graphs represent means of three independent experiments.

Activation of TRAIL death pathway has been shown in viral infection [24], [25]. Our data showed that HCV infection indeed induced TRAIL and its receptors DR4 and DR5 (Figure 5A). Silencing of TRAIL inhibited the induction of ISG12a and blocked apoptosis of viral-infected cells (Figure 5B and 5C), indicating that HCV infection triggers apoptosis of HLCZ01 cells through TRAIL-mediated pathway.

Inhibition of ISG12a expression prevents the sensitization to etoposide-induced apoptosis [26]. In our study, HCV infection highly induced ISG12a expression (Figure 2B). To assess the impact of ISG12a on apoptosis of HLCZ01 cells induced by HCV infection, we used shRNA constructs pSilencer-ISG12a shRNA designed to silence ISG12a in HLCZ01 cells (Figure 5D). The efficiency of ISG12a shRNA knockdown was confirmed (Figure 5D). As expected, silencing of ISG12a reduced PARP inactivation as assessed by the appearance of the cleaved fragments in HCV-infected HLCZ01 cells (Figure 5E). Flow cytometry revealed that ISG12a knockdown prevented HCV-infected HLCZ01 cells from apoptosis compared with the control (Figure 5F). Collectively, these results highlighted that HCV infection triggers apoptosis of viral infected hepatocytes through ISG12a relying on TRAIL-mediated pathway.

### MicroRNA-942 (miR-942) regulates HCV-induced apoptosis of human hepatocytes through targeting ISG12a

One recent report suggests that microRNA can regulate ISG expression [27]. To determine the mechanisms implicated in the regulation of ISG12a, we performed a bioinformatics search for putative microRNA targets of ISG12a. 3′UTR of human ISG12a contains region that matches the seed sequence of human miR-942. Moreover, miR-942 was markedly downregulated in HCV-infected HLCZ01 cells verse naïve HLCZ01 cells (Figure S3A). MiR-942 was inversely correlated with ISG12a expression in liver biopsies of chronic HCV-infected patients (Figure S3B). So we proposed that ISG12a is a target of miR-942. To verify this hypothesis, we cloned 3′UTR of ISG12a into downstream of the luciferase ORF of pGL3 control vector. The reporter construct pGL3-ISG12aUTR and pcDNA3.1-miR-942 were transfected into HLCZ01 cells. Forced expression of miR-942 in HLCZ01 cells, confirmed by real-time PCR (Figure S3C), markedly reduced luciferase activity (Figure S3D). When we performed luciferase assays using a plasmid harboring ISG12a3′UTR(Mut), where the binding sites for miR-942 were inactivated, we did not observe inhibitory effect of miR-942 on luciferase activity (Figure S3D). To examine whether miR-942 affects ISG12a expression in HLCZ01 cells, we examined the effect of forced expression of miR-942 on the level of ISG12a in HLCZ01 cells. Overexpression of miR-942 upon transfection significantly decreased ISG12a level in comparison with the control (Figure S3E). Knockdown of miR-942 by anti-miR-942 in HLCZ01 cells, which was confirmed by quantitative real-time PCR (Figure S3F), increased ISG12a protein level (Figure S3G). The expression of miR-942 expression was downregulated in viral-infected cells at earlier phase and return to normal level at late phase. The expression of ISG12a increased too fast to be inhibited by miR-942 at later phase. All the data supported that the ISG12a is a direct targeted gene of miR-942.

**Figure 6:**
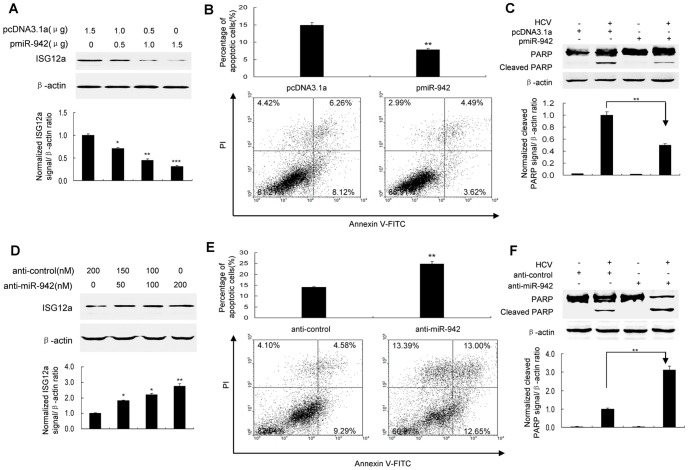
MiR-942 modulates HCV-induced apoptosis of human hepatocytes via targeting ISG12a. (**A**) Forced expression of miR-942 in HCV-infected HLCZ01 cells reduced the level of ISG12a. HLCZ01 cells were transfected with pcDNA3.1-miR-942 (pmiR-942). ISG12a was examined by western blot. β–actin was used as control. (**B/C**) Forced expression of miR-942 in HCV-infected HLCZ01 cells caused a marked decrease in apoptosis induction as determined by flow cytometry and PARP cleavage. HLCZ01 cells were transfected with pcDNA3.1-miR-942 and infected by HCV for 9 days. (B) The cells were examined by flow cytometry. (C) Inactivation of PARP was determined by western blot. (**D–F**) Knockdown of miR-942 expression in HLCZ01 cells by anti-miR-942 increased ISG12a expression and enhanced HCV-induced apoptosis. HLCZ01 cells were transfected by anti-miR-942 and infected by HCV for 9 days. (D) ISG12a was examined by western blot. β–actin was used as control. (E/F) Knockdown of miR-942 expression in HLCZ01 cells by anti-miR-942 in HCV-infected HLCZ01 cells caused an increase in apoptosis induction as determined by flow cytometry (E) and PARP cleavage (F). If not stated otherwise bar graphs represent means of three independent experiments.

To examined the effect of miR-942 on ISG12a expression and HCV-induced apoptosis of HLCZ01 cells, we transfected pcDNA3.1-miR-942 into HLCZ01 cells and detected the level of ISG12a and apoptosis of HLCZ01 in response to HCV infection. Forced expression of miR-942 in HLCZ01 cells markedly reduced ISG12a (Figure 6A and Figure S3E) and caused a strong decrease in apoptosis induction determined by flow cytometry (Figure 6B) and PARP cleavage (Figure 6C). Conversely, silencing miR-942 by anti-miR-942 in HLCZ01 cells increased ISG12a expression in HCV-infected cells (Figure 6D and Figure S3G) and enhanced apoptosis triggered by HCV infection measured by flow cytometry (Figure 6E) and PARP cleavage (Figure 6F). The data suggested that miR-942 regulates HCV-induced apoptosis of human hepatocytes by targeting ISG12a.

### Induction of Noxa by HCV infection contributes to ISG12a-mediated apoptosis

**Figure 7:**
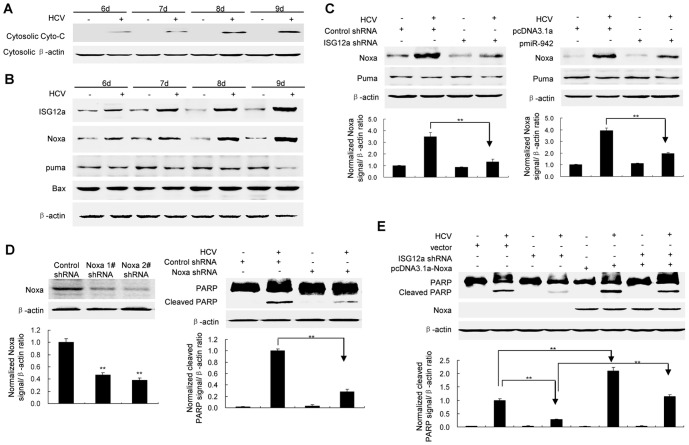
Induction of Noxa contributes to ISG12a-mediated apoptosis. (**A**) Detection of cytosolic cytochrome c. HLCZ01 cells were infected with HCV at MOI of 0.1. Cytosolic cytochrome c was detected by western blot. (**B**) The cells were treated as described in part A. ISG12a, Noxa, Puma and Bax were detected by western blot. (**C**) Silencing of ISG12a or overexpression of miR-942 decreased Noxa expression. HLCZ01 cells were transfected by pcDNA3.1-ISG12a shRNA or pcDNA3.1-miR-942, followed with HCV infection at MOI of 0.1 for 9 days. Noxa and Puma were detected by western blot receptively. (**D**) Silencing of Noxa inhibited HCV-induced apoptosis. HLCZ01 cells were transfected by pcDNA3.1-Noxa shRNA or control vector, followed with HCV infection at MOI of 0.1 for 9 days. Noxa and PARP cleavage was detected by western blot. (**E**) Noxa overexpression reversed the inhibition of apoptosis of ISG12a-silenced HLCZ01 cells. HLCZ01 cells were transfected by ISG12a shRNA or control vector, followed with pcDNA3.1-Noxa transfection. Then the cells were infected with HCV at MOI of 0.1 for 9 days. The cells were collected and PARP cleavage was detected by western blot. If not stated otherwise blots are representative of three independent experiments.

To determine the apoptotic pathway involved in HCV-induced apoptosis in HLCZ01 cells, we examined cytochrome C in HCV-infected HLCZ01 cells. Cytosolic cytochrome C was observed in cells with HCV infection, indicating that HCV infection activates mitochondrial-dependent apoptotic pathway (Figure 7A). Mitochondrial-dependent apoptotic pathway is regulated by BH-3 only proteins of Bcl-2 family. Ectopic expression of Noxa enhances viral-induced apoptosis, typified by enhanced cytochrome c release from mitochondrial to the cytosolic fraction [28]. HCV infection induced Noxa and ISG12a in HLCZ01 cells although the levels of Puma and Bax were not changed in viral-infected HLCZ01 cells (Figure 7B). Moreover, silencing of ISG12a or overexpression of miR-942 decreased the expression of Noxa while it had no effect on Puma (Figure 7C). Silencing of Noxa by shRNA inhibited HCV-induced apoptosis (Figure 7D). Noxa overexpression reversed the inhibition of apoptosis of ISG12a-silenced HLCZ01 cells (Figure 7E). These data suggested that induction of Noxa by HCV contributes to ISG12a-mediated apoptosis.

## Discussion

Various hepatic cell culture systems including hepatoma cell lines Huh7 and Huh7.5 have been used to explore hepatic innate immune response to HCV. However, these poor-differentiated hepatoma cells differ from primary human hepatocytes and well-differentiated HCC cells, raising doubts as to the *in vivo* relevance of the widely used in vitro system. Moreover, Huh7.5 cells may not mount an intact innate antiviral response to HCV infection, so it is difficult to explore the virus and host cells interaction. Although primary human hepatocytes closely mimic the natural target cell of HCV and are the best model of choice for the study of pathogenesis of chronic C *in vitro* [14], [29], the use of primary human hepatocytes is hampered by the limited availability and unpredictable variability of human liver.

To explore the interaction between HCV and host cells, we used a newly established HCC cell line HLCZ01 in this study. When HLCZ01 cells were infected with JFH1 virus, HCV RNA was readily detected 24 hours postinfection. The replication efficiency of JFH1 virus in this novel culture system was comparable to Huh7.5. At day 9 postinfection, many cells died. The survival cells still supported viral replication and finally reached pretty high efficiency of viral replication. It has been demonstrated that Huh7.5 cell line possess an inactivating mutation in RIG-I [12], an important component for IFN response via virus-related dsRNA-sensing machinery and thus lacks a functional RIG-I signaling pathway. JFH1 virus fails to induce IFN and ISGs expression in this cell line as demonstrated in our and other studies [16], [30]. However, the same viruses are able to induce the IFN-β and ISGs expression in current cell culture system and induction of IFN-β by viral infection in HLCZ01 cells depends on RIG-I signaling pathway. The intact innate immune system evidenced by induction of IFN-β and apoptosis in HLCZ01 cells in response to HCV infection may contribute to slightly low viral replication efficiency in HLCZ01 compared to Huh7.5 cells.

It has been reported that another HCV-2a strain induces hepatocellular apoptosis [31]. The current study demonstrated that RIG-I and subsequent IRF-3 regulate IFN-β expression and are responsible for HCV-induced apoptosis of HLCZ01 cells via activation of TRAIL-mediated pathway. The intact innate antiviral system in HLCZ01 cells will allow us to further study the molecular details on how HCV induces innate antiviral responses in human hepatocytes.

To identify the mechanisms of how HCV infection causes apoptosis in HLCZ01 cells, we analyzed the gene expression profile in HCV-infected HLCZ01 cells verse naïve cells. We found that ISG12a was consistently highly expressed in HCV-infected HLCZ01 cells compared to naïve cells. It has been reported that ISG12a mediates antiviral effects against different neurotropic viruses [27]. Our data showed that silencing of ISG12a prevented HCV-infected HLCZ01 cells from apoptosis compared with the control. Apoptosis induction by HCV infection in HLCZ01 cells involves ISG12a which relies on TRAIL-mediated signaling. Collectively, these data supported that HCV infection triggers apoptosis of viral-infected hepatocytes through ISG12a. Our study implicated that ISG12a is a contributing regulator of TRAIL-induced apoptosis, which enhances the antiviral activities of type I IFN.

To determine the mechanisms implicated in the regulation of ISG12a in HCV-infected hepatocytes, we assessed the microRNA expression profile in HCV-infected HLCZ01 cells. Our data demonstrate that miR-942 was reduced in HCV-infected cells and ISG12a was a targeted gene of miR-942. Forced expression of miR-942 in HLCZ01 cells markedly reduced ISG12a level and caused a marked decrease in HCV-induced apoptosis. However, silencing of miR-942 expression by anti-miR-942 increased the expression of ISG12a in HLCZ01 cells and subsequently enhanced apoptosis triggered by HCV infection. All the data indicated that miR-942 modulates HCV-induced apoptosis of hepatocytes by targeting ISG12a.

Current study demonstrated the mechanism by which HCV infection induces cytopathic and noncytopathic antiviral response in human hepatocytes. Apoptosis of viral-infected hepatocytes is an efficient way to eliminate viral infection. Induction of IFN by HCV infection can protect neighboring cells from new rounds of viral infection. To establish persistent infection in the presence of intracellular innate immune response, it is logical for the virus to develop various evasion strategies by which the virus blocks the antiviral response in host cells. Several studies have demonstrated how HCV inactivates intracellular innate response. HCV NS34A protease interferes with RIG-I signaling pathway and cleaves MAVS, thereby blocking IFN-β production [32]–[35]. The studies were performed through experiments using HCV replicon and hepatoma cell transfection systems. It is necessary to use our novel HCV culture system to explore the interaction between the virus and host cells.

In summary, our study has demonstrated that human hepatocytes have intact innate immune response evidenced by induction of IFN-β and apoptosis in HLCZ01 cells with HCV infection. RIG-I plays a key role in the induction of IFN and apoptosis of hepatocytes in response to HCV infection. Apoptosis triggered by HCV infection in HLCZ01 cells involves ISG12a which relies on TRAIL-mediated pathway. MiR-942 modulates HCV-induced apoptosis of human hepatocytes through targeting ISG12a. Induction of Noxa by HCV infection contributes to ISG12a-mediated apoptosis. These findings reveal a novel mechanism by which human hepatocytes respond to HCV infection. The *in vitro* system for the complete replication of infectious HCV in HLCZ01 cells will facilitate the molecular analysis of infectious virus-host interactions.

## Supporting Information

Figure S1 **Establishment of a new hepatoma cell line HLCZ01.** (**A**) H&E section of the moderated-differentiated hepatocellular carcinoma from a male patient. (**B**) HLCZ01 cells express liver-specific proteins. Protein was isolated from HLCZ01, primary human hepatocytes (PHH), Huh7.5 and CHO cells. Human α1-antitrypsin (AAT) and albumin (ALB) protein was detected by western blot. (**C**) HLCZ01 cells express CD81 protein. HLCZ01 and Huh7.5 cells were harvested for immunostaining using mouse monoclonal anti-human CD81. DAPI was used for nuclei counterstaining. Identical setting was maintained for images capture.
(TIF)

Figure S2 **HCV infection triggers apoptosis of HLCZ01 cells.** HLCZ01 cells were incubated with JFH1 virus at MOI of 0.1 for 9 days. The cells were harvested and stained with DAPI (blue) and NS5A (red). Apparent nuclear condensation and fragmentation were seen in HLCZ01 cells infected with HCV. The white arrows represent apoptotic cells.
(TIF)

Figure S3 **MR-942 directly targets 3′UTR of ISG12a.** (**A**) miR-942 is downregulated in HCV-infected HLCZ01 cells verse naïve HLCZ01 cells. HLCZ01 cells were infected by HCV and NDV at MOI of 0.1 (Losota). MiR-942 was examined by real-time PCR. The expression of miR-942 was normalized with U6. (**B**) MiR-942 is inversely correlated with ISG12a expression in liver tissues of chronic HCV-infected patients. Total cellular RNA was isolated from liver tissues of chronic HCV-infected patients. The expression of miR-942 and ISG12a was examined by real-time PCR and normalized with U6 and GAPDH respectively. (**C/D**) pGL3-ISG12aUTR luciferase construct containing wild type or mutated (Mut) ISG12a 3′UTR was transfected into HLCZ01 cells together with pcDNA3.1-miR-942. Expression of miR-942 was normalized with U6 (C). Relative firefly luciferase expression was standardized to a transfection control. The reporter assays were performed in triplicate (D). (**E**) The effect of miR-942 forced expression on ISG12a level in viral-infected HLCZ01 cells. HCV-infected HLCZ01 cells were transfected with pcDNA3.1-miR-942. ISG12a was examined by real-time PCR and normalized with GAPDH. (**F/G**) Knockdown of miR-942 by anti-miR-942 increased ISG12a level in HLCZ01 cells. Anti-miR-942 was delivered into HLCZ01 cells. MiR-942 (F) or ISG12a (G) was examined by real-time PCR. The expression of miR-942 or ISG12a was normalized with U6 or GAPDH respectively. The data represented the means of 3 independent experiments.
(TIF)
