# Supplementary figures and images for: MiR-942 Mediates Hepatitis C Virus-Induced Apoptosis via Regulation of ISG12a
Source: PLoS One. 2014 Apr 11;9(4):e94501. doi: 10.1371/journal.pone.0094501 (PMC3984147; doi:10.1371/journal.pone.0094501)

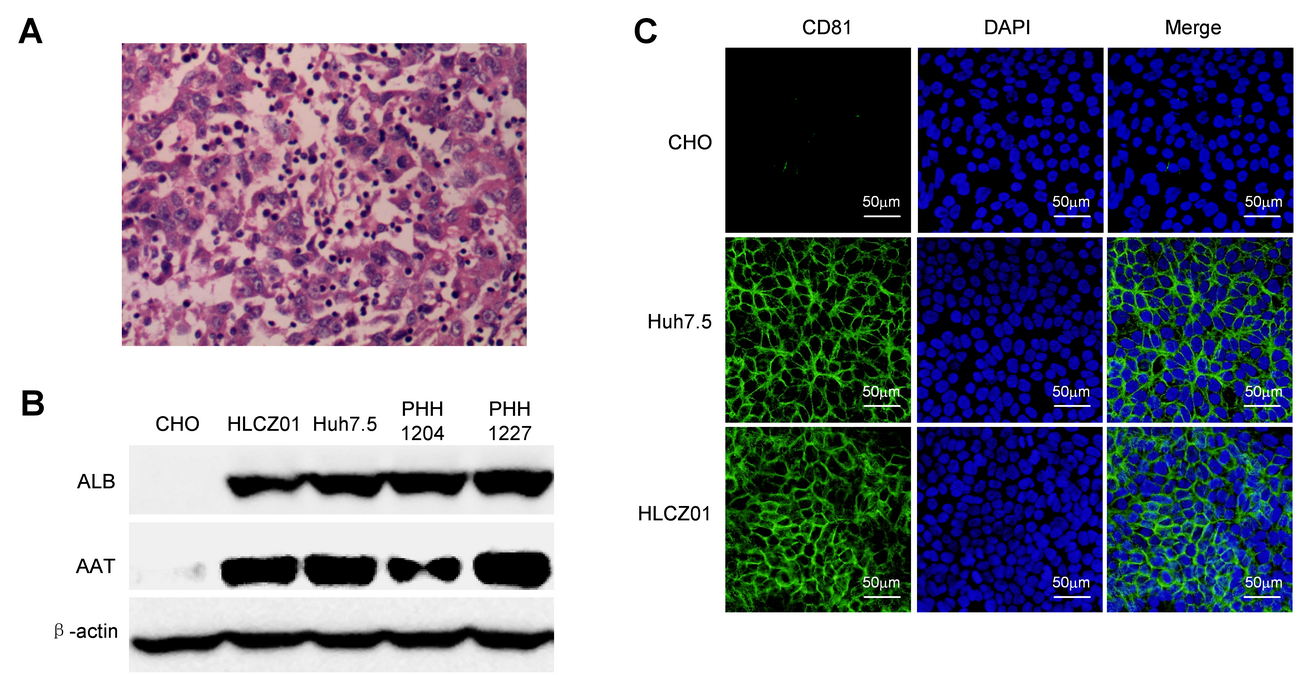

Supplement: Figure S1 — Establishment of a new hepatoma cell line HLCZ01. (A) H&E section of the moderated-differentiated hepatocellular carcinoma from a male patient. (B) HLCZ01 cells express liver-specific proteins. Protein was isolated from HLCZ01, primary human hepatocytes (PHH), Huh7.5 and CHO cells. Human α1-antitrypsin (AAT) and albumin (ALB) protein was detected by western blot. (C) HLCZ01 cells express CD81 protein. HLCZ01 and Huh7.5 cells were harvested for immunostaining using mouse monoclonal anti-human CD81. DAPI was used for nuclei counterstaining. Identical setting was maintained for images capture. (TIF) [file pone.0094501.s001.tif]

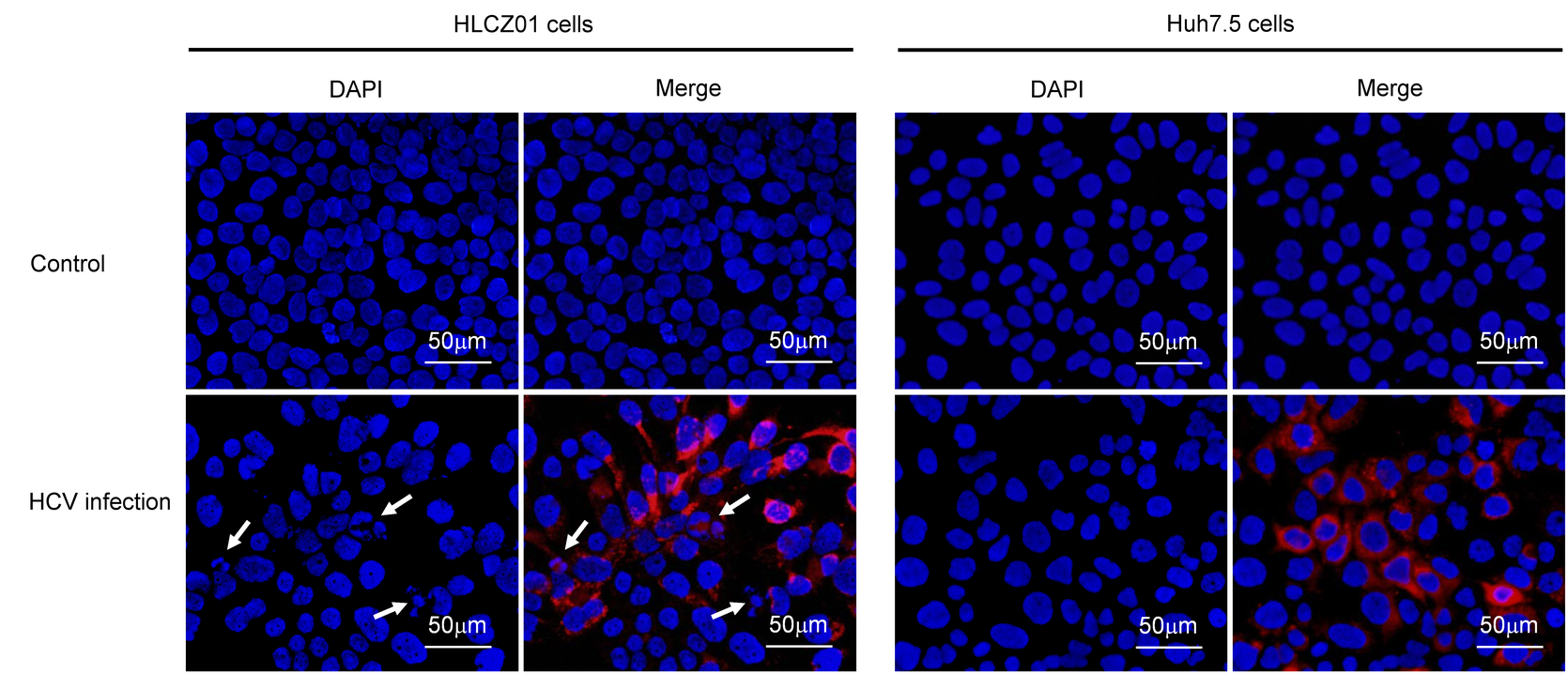

Supplement: Figure S2 — HCV infection triggers apoptosis of HLCZ01 cells. HLCZ01 cells were incubated with JFH1 virus at MOI of 0.1 for 9 days. The cells were harvested and stained with DAPI (blue) and NS5A (red). Apparent nuclear condensation and fragmentation were seen in HLCZ01 cells infected with HCV. The white arrows represent apoptotic cells. (TIF) [file pone.0094501.s002.tif]

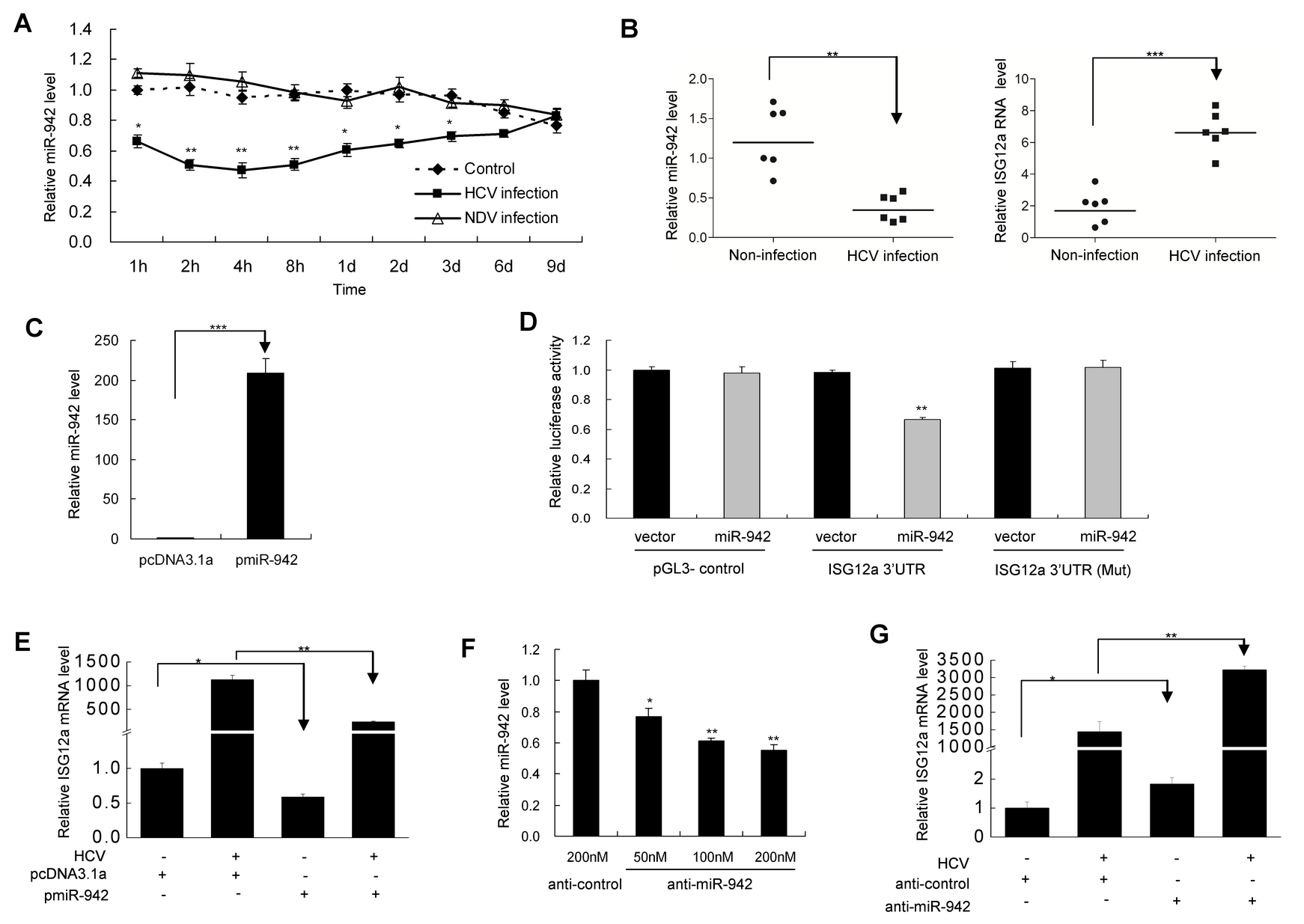

Supplement: Figure S3 — MR-942 directly targets 3′UTR of ISG12a. (A) miR-942 is downregulated in HCV-infected HLCZ01 cells verse naïve HLCZ01 cells. HLCZ01 cells were infected by HCV and NDV at MOI of 0.1 (Losota). MiR-942 was examined by real-time PCR. The expression of miR-942 was normalized with U6. (B) MiR-942 is inversely correlated with ISG12a expression in liver tissues of chronic HCV-infected patients. Total cellular RNA was isolated from liver tissues of chronic HCV-infected patients. The expression of miR-942 and ISG12a was examined by real-time PCR and normalized with U6 and GAPDH respectively. (C/D) pGL3-ISG12aUTR luciferase construct containing wild type or mutated (Mut) ISG12a 3′UTR was transfected into HLCZ01 cells together with pcDNA3.1-miR-942. Expression of miR-942 was normalized with U6 (C). Relative firefly luciferase expression was standardized to a transfection control. The reporter assays were performed in triplicate (D). (E) The effect of miR-942 forced expression on ISG12a level in viral-infected HLCZ01 cells. HCV-infected HLCZ01 cells were transfected with pcDNA3.1-miR-942. ISG12a was examined by real-time PCR and normalized with GAPDH. (F/G) Knockdown of miR-942 by anti-miR-942 increased ISG12a level in HLCZ01 cells. Anti-miR-942 was delivered into HLCZ01 cells. MiR-942 (F) or ISG12a (G) was examined by real-time PCR. The expression of miR-942 or ISG12a was normalized with U6 or GAPDH respectively. The data represented the means of 3 independent experiments. (TIF) [file pone.0094501.s003.tif]
